# Identification of hypoxia- and immune-related biomarkers in patients with ischemic stroke

**DOI:** 10.1016/j.heliyon.2024.e25866

**Published:** 2024-02-07

**Authors:** Haofuzi Zhang, Jidong Sun, Peng Zou, Yutao Huang, Qiuzi Yang, Zhuoyuan Zhang, Peng Luo, Xiaofan Jiang

**Affiliations:** a Department of Neurosurgery, Xijing Hospital, Fourth Military Medical University, Xi'an, China; b Biochemistry and Molecular Biology, College of Life Science, Northwest University, Xi'an, China

**Keywords:** Ischemic stroke, Hub gene, Hypoxia-related genes, Immune microenvironment, Immune cell infiltration

## Abstract

**Background:**

The immune microenvironment and hypoxia play crucial roles in the pathophysiology of ischemic stroke (IS). Hence, in this study, we aimed to identify hypoxia- and immune-related biomarkers in IS.

**Methods:**

The IS microarray dataset GSE16561 was examined to determine differentially expressed genes (DEGs) utilizing bioinformatics-based analysis. The intersection of hypoxia-related genes and DEGs was conducted to identify differentially expressed hypoxia-related genes (DEHRGs). Then, using weighted correlation network analysis (WGCNA), all of the genes in GSE16561 dataset were examined to create a co-expression network, and module–clinical trait correlations were examined for the purpose of examining the genes linked to immune cells. The immune-related DEHRGs were submitted to gene ontology (GO) and Kyoto Encyclopedia of Genes and Genomes (KEGG) enrichment analyses. A protein–protein interaction (PPI) network was constructed by Cytoscape plugin MCODE, in order to extract hub genes. The miRNet was used to predict hub gene-related transcription factors (TFs) and miRNAs. Finally, a diagnostic model was developed by least absolute shrinkage and selection operator (LASSO) logistic regression.

**Results:**

Between the control and IS samples, 4171 DEGs were found. Thereafter, the intersection of hypoxia-related genes and DEGs was conducted to obtain 45 DEHRGs. Ten significantly differentially infiltrated immune cells were found—namely, CD56dim natural killer cells, activated CD8 T cells, activated dendritic cells, activated B cells, central memory CD8 T cells, effector memory CD8 T cells, natural killer cells, gamma delta T cells, plasmacytoid dendritic cells, and neutrophils—between IS and control samples. Subsequently, we identified 27 immune-related DEHRGs through the intersection of DEHRGs and genes in important modules of WGCNA. The immune-related DEHRGs were primarily enriched in response to hypoxia, cellular polysaccharide metabolic process, response to decreased oxygen levels, polysaccharide metabolic process, lipid and atherosclerosis, and HIF−1 signaling pathway H. Using MCODE, FOS, DDIT3, DUSP1, and NFIL3 were found to be hub genes. In the validation cohort and training set, the AUC values of the diagnostic model were 0.9188034 and 0.9395085, respectively.

**Conclusion:**

In brief, we identified and validated four hub genes—FOS, DDIT3, DUSP1, and NFIL3—which might be involved in the pathological development of IS, potentially providing novel perspectives for the diagnosis and treatment of IS.

## Graphical abstract

**Figure:**
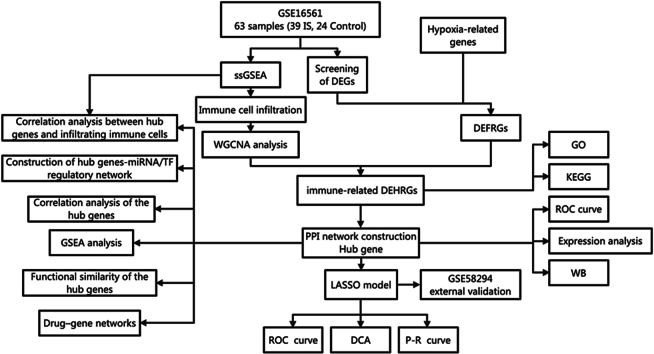

## 1 Introduction

The most frequent cause of disability and the second leading cause of death worldwide is stroke [1]. Around 15 million people worldwide are affected every year, comprising 5 million fatalities and an additional 5 million individuals with permanent physical impairments [2]. New research has shown that the incidence of stroke is on the rise, and a quarter of people around the world have experienced stroke in their lifetime [3]. The aging population and the burden of cumulative risk factors have led to an increased probability of having a stroke that lasts a lifetime. Furthermore, the improving socio-economic position of developing nations has resulted in an increased prevalence of stroke risk factors in young people [4]. Stroke is essentially divided into ischemic or hemorrhagic stroke. A blood vessel obstruction that lowers the supply of blood to specific parts of the brain causes an ischemic stroke (IS), while cerebral (or subarachnoid) hemorrhage caused by cerebral vascular rupture results in hemorrhagic stroke [5,6]. IS is the most prevalent type of stroke globally, as has been confirmed by ample research [7,8]. Previous studies have mainly focused on the mechanism of neuronal injury following cerebral ischemia, and selected drug intervention targets for these injury mechanisms. Although these drugs have achieved significant brain protective effects in animal experiments, their therapeutic effects in clinical application are still not ideal [9]. In order to better treat IS patients, we still need to develop therapeutic targets and diagnostic markers for IS.

A significant advance in IS treatment involves the post-stroke immune response. The key elements of the central nervous system (CNS)-infarct-surrounding environment are glial cells (e.g., oligodendrocytes, microglia, and astrocytes), which are related to post-stroke immune regulation [10]. Glial cells after a stroke control neuroinflammation, according to various studies [11,12]. These cells have the ability to integrate and recognize signs of neuronal injury, draw immune cells to the site of the stroke, interact with other immune cells, and release cytokines to influence their status. However, at present, there exists no effective treatment for immune response after IS. Tissue oxygen content plays a crucial role in maintaining the function of normal cells, regulating their development. The CNS neurons are especially vulnerable to hypoxia. Hypoxia can alter mitochondrial activity and oxygen metabolism, which can damage the structural and functional properties of neurons, even leading to their death [13,14]. The brain requires a timely and adequate supply of energy and oxygen, as it is the body's most vulnerable organ. A lack of blood flow causes a chain of pathological events that will eventually result in IS. These events cause a significant number of nerve cells to die in the ischemic area and impair nerve function within a few hours. Following cerebral ischemia, mitochondrial dysfunction, bioenergy failure, inflammation, neuroexcitotoxicity, protein folding mistakes, and neuronal apoptosis may take place, leading to the rapid development of penumbra in ischemic brain tissue, until irreversible infarction occurs [15]. IS is a major source of hypoxic brain stress [16]. A protein known as hypoxia-inducible factor-1 (HIF-1) controls the expression of over seven hundred target genes that regulate pathological and adaptive processes, making it one of the key regulators of the cell response to hypoxia [[17], [18], [19]]. By targeting downstream genes and controlling numerous signaling pathways, HIF-1α mediates an endogenous adaptive program under hypoxic conditions, which has been linked to the pathophysiology of a number of neurological disorders, especially IS [20]. A recent clinical investigation has revealed increased HIF-1α in serum 48 h after acute IS. Such an elevated concentration is expected to be a predictor of stroke prognosis [21]. HIF-1α is expressed not merely in neurons, but likewise in microglia, astrocytes, and endothelial cells, having a cell type-specific role [22,23]. In neurons, the function of HIF-1α has attracted the interest of many researchers.

The above researches indicate that hypoxia and the immune response play important roles in IS. Our research intends to investigate gene expression alterations in the pathophysiology of IS, in order to develop new possible diagnostic biomarkers. Collectively, these findings may provide novel insights into the molecular immune mechanisms and reveal potential novel approaches for the treatment of IS.

## 2 Materials and methods

### 2.1 Data source

The expression matrix of GSE58294 and GSE16561 datasets was acquired from the Gene Expression Omnibus (GEO) database (https://www.ncbi.nlm.nih.gov/geo/) in this study. The GSE16561 dataset (GPL6883 platform) including 39 IS samples and 24 control samples was employed as the training set. The GSE58294 dataset (GPL570 platform) including 23 IS samples and 69 control samples was used as the external validation set. Hypoxia-related genes were acquired from the HALLMARK_HYPOXIA gene set in the MSigDB database.

### 2.2 Screening of differentially expressed genes (DEGs)

The DEGs between the IS and control samples were screened using the limma package [24] under the criteria of adj.P < 0.05 in the GSE16561 dataset. The results were displayed as a volcano plot and heatmap utilizing the ggplot2 and pheatmap R packages, respectively. The intersection between DEGs and hypoxia-related genes was identified by employing the VennDiagram R package [25], and were described as differentially expressed hypoxia-related genes (DEHRGs) for subsequent analysis.

### 2.3 Immune infiltration analysis

Single-sample gene set enrichment analysis (ssGSEA) was employed to estimate the relative infiltration scores of 28 immune cell types in the IS and control samples through the GSVA package [26]. We then contrasted the immune cell infiltration by Wilcoxon test and p value was corrected by Bonferroni method.

### 2.4 Weighted gene co-expression network analysis (WGCNA) analysis

The WGCNA R package was used to conduct WGCNA based on the expression data of GSE16561 dataset [27]. First, a sample clustering tree was constructed to remove outliers based on cutHeight. The soft-thresholding power β was defined to ensure a standard scale-free network. The dynamic pruning approach was then employed to find co-expression modules. By WGCNA, the modules most relevant to the differentially infiltrating immune cells could be identified. Therefore, the intersection of genes and DEHRGs in important modules was carried out in order to define immune-related DEHRGs.

### 2.5 Functional enrichment analysis

In order to explore the function of immune-related DEHRGs, Gene Ontology (GO) and the Kyoto Encyclopedia of Genes and Genomes (KEGG) enrichment analysis were conducted by clusterProfiler package [28]. GO is a powerful tool to analyze the molecular function (MF), cellular component (CC), and biological function (BP) of immune-related DEHRGs.

### 2.6 Protein-protein interaction (PPI) network construction

The STRING database was then used to build the PPI network [29]. Subsequently, we used the Molecular Complex Detection (MCODE) plug-in in Cytoscape software to screen hub genes. The semantic similarities of gene classes were calculated using the GOSemSim package [30], and hub gene associations were examined using the Corrplot package.

### 2.7 Correlation analysis between hub genes and infiltrating immune cells

The associations among the 28 infiltrating immune cells and hub gene expression were examined. The associations among differently invading immune cells and hub genes were displayed using the corrplot R package.

### 2.8 Small-molecule drug prediction and GSEA analysis

The Drug Gene Interaction Database (DGIdb) database was used to mine the potential drugs targeting hub genes [31]. The Cytoscape software was used to visualize the determined target network. The potential roles of hub genes were analyzed by GSEA enrichment analysis. The clusterprofile package was employed to perform GSEA of the hub genes. And C2.cp.kegg.v7.0.symbols.gmt was used as the reference gene set for GSEA.

### 2.9 Construction of the regulatory network

In our investigation, the miRNet database (https://www.mirnet.ca/) was chosen [32] to anticipate the regulatory link between mircoRNAs (miRNAs)/transcription factors (TFs) and hub genes. Using Cytoscape, the miRNA/TF–hub gene network was created. The hub genes are represented by blue nodes in the network, TFs are represented by purple nodes, and miRNAs are represented by orange nodes.

### 2.10 Receiver operating characteristic (ROC) curve analysis

ROC curves were plotted to assess the diagnostic power of hub genes using the pROC package [33]. The hub genes were thought to have great specificity and sensitivity for separating IS from control when the area under the curve (AUC) value was greater than 0.7. A boxplot was used to depict the hub gene expressions in GSE16561 between control and IS.

### 2.11 Establishment of a LASSO model

On the basis of the hub gene expression profiles, the least absolute shrinkage and selection operator (LASSO) algorithm was employed to develop the IS diagnostic model, utilizing the glmnet R package. The accuracy and the effectiveness of the model were assessed according to the P–R curves, ROC curves, and decision curve analysis (DCA).

### 2.12 Mouse MCAO model

For at least 7 days prior to the study, male C57BL/6 mice (aged 10–12 weeks, weighing 25–28 g) were housed in cages (six mice per cage) in an air-conditioned room at a constant temperature of about 27 °C and with a 12 h light/dark cycle. The mice were acquired from the Experimental Center of the Fourth Military Medical University. Entire animal research was conducted in compliance with the Fourth Military Medical University Committee on Animal Care (No. 20210419) and National Institutes of Health Guidelines for the Care and Use of Laboratory Animals. C57BL/6 mice were fasted for 12 h and drank freely before the operation. The anesthetized mice were inhaled with a small animal anesthesia machine, the scalp was cut to expose the muscle tissue, and a laser speckle blood flow imaging system was fixed at the projection of the middle cerebral artery. The internal carotid artery, right common carotid artery, and external carotid artery were exposed layer-by-layer in the neck's median incision while the mice were fixed in the supine posture on a 37 °C thermostatic plate. The external carotid artery and common carotid artery were tied together, and a thread plug with a depth of about 10 mm was placed about 5 mm from the common carotid artery's bifurcation. The laser speckle flow imaging system was employed to verify whether the local cerebral blood flow on the embolic side of the middle cerebral artery decreased below 30% of the basic value. After 1 h, the lead bolt was pulled out and tissue perfusion was restored. Again, the laser speckle flow imaging system was employed to verify whether the local cerebral blood flow on the embolic side of the middle cerebral artery recovered to more than 80% of the basic value. We utilized a total of six mice, which include three in the control group and three in the MCAO group, and the control group underwent sham surgery.

### 2.13 Antibodies

A primary antibody against FOS (#GB12069, mouse mAb) was obtained from Servicebio (Wuhan, China). Antibodies against DDIT3 (#15204-1-AP, rabbit mAb), NFIL3 (#11773-1-AP, rabbit mAb), and Beta-Tubulin (#10094-1-AP, rabbit mAb) were acquired from Proteintech (Rosemont, IL, USA). Antibody against DUSP1 (#AF6750, rabbit mAb) was obtained from Beyotime Biotechnology (Shanghai, China). For immunoblotting, HRP-conjugated goat anti-rabbit (#sc-2004) and goat anti-mouse (#sc-2005) secondary antibodies (Santa Cruz Biotechnology, CA, USA) were employed. For immunohistochemistry, HRP-conjugated goat anti-rabbit/mouse IgG H&L (#PV-6000D) secondary antibodies (ZSGB-bio, Beijing, China) were employed.

### 2.14 Western Blot analysis

Following the different treatments, brain tissue from the damage location was lysed in a solution containing PhosSTOP phosphatase inhibitor (#5892970001) tablets and protease inhibitor (#4906845001; Roche Applied Bioscience, Indianapolis, IN, USA). A BCA protein kit was employed to measure the amount of protein in the supernatant. On 10–15% and 10% SDS-PAGE gels, the proteins were separated, then transferred to nitrocellulose membranes (Thermo Fisher Scientific). The membranes were cut in accordance with the specific molecular weights and pre-stained protein ladders (#26617, Thermo Fisher Scientific). The membranes were incubated with the appropriate primary antibodies overnight at 4 °C after being soaked in 5% non-fat milk in Tris-buffered saline and 0.05 percent Tween 20 (TBST) at room temperature (DUSP1, 1:500 dilution; FOS, 1:500 dilution; NFIL3, 1:500 dilution; DDIT3, 1:500 dilution; Beta-Tubulin, 1:5000 dilution). After being washed with TBST, the membranes were incubated with secondary antibodies that had been diluted in blocking buffer for 1 h at room temperature. A goat anti-rabbit secondary antibody was used to precisely identify the DDIT3, DUSP1, beta-tubulin, and NFIL3 antibodies. A goat anti-mouse antibody was used to selectively identify the FOS antibody. With the use of SuperSignal West Pico Chemiluminescent Substrate, the immunoreactivity was determined (Thermo Fisher Scientific). The ImageJ image analysis program was used (National Institutes of Health, MA, USA) to quantify the optical densities of the bands.

### 2.15 Immunohistochemistry (IHC)

The brain was harvested following standard perfusion and then fixed with 4% paraformaldehyde for a span of 8–10 h. Subsequently, the brain tissue was dehydrated utilizing 30% ethanol for a full 24-h period. Then the brain tissue was conventionally embedded using a paraffin-embedding machine. Post-embedding, the paraffin-infused tissue was sectioned into 1 μm thicknesses using a slicer. A heat-induced epitope retrieval approach was employed to retrieve the slides, which were subsequently blocked with 5% goat serum (#SP-9001, ZSGB-bio, Beijing, China) for 10 min. The primary antibody was then added, and the mixture was gently shaken and incubated at 4 °C for an entire night. The secondary antibody working solution was then added and incubated for 10 min at room temperature following three PBS washes. Streptavidin/HRP working solution (#SP-9001, ZSGB-bio, Beijing, China) was added to the slides after three PBS washes, then left to incubate for 10 min at room temperature. Fresh DAB working solution (#SP-9001, ZSGB-bio, Beijing, China) was added and incubated for 5 min at room temperature after three PBS washes. The slides were cleaned, stained with hematoxylin, and then examined using inverted fluorescence microscopy (Olympus IX73).

### 2.16 Statistical analysis

The R programming language was employed for all the bioinformatics analyses, and the Wilcoxon test was employed to compare the data from various groups. A *p*-value less than 0.05 was deemed statistically significant, unless otherwise stated. Our code and more details are available on our project page (https://github.com/zhanghaofuzi/Identification-of-hypoxia--and-immune-related-biomarkers-in-patients-with-ischemic-stroke).

## 3 Results

### 3.1 Screening of DEGs

**Fig. 1:**
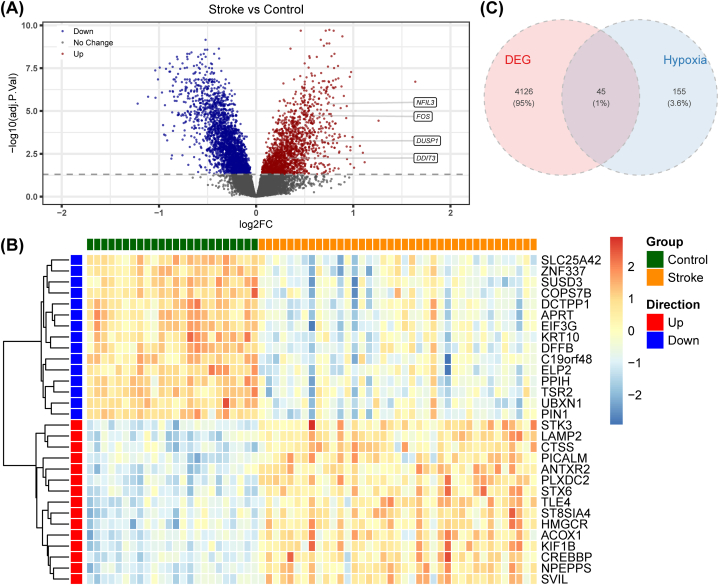
Identification of DEGs in IS: (**a**) Volcano plot of DEGs among IS and normal sample; (**b**) Heatmap of Top 15 up- and down-regulated DEGs between normal and IS sample; and (**c**) Wayne diagram of DEFRGs.

To explore the extent of differences in gene expression between the IS and normal states, we mined DEGs. In total, 4171 DEGs between IS and control groups were screened, including 1645 up-regulated genes and 2526 down-regulated genes (Fig. 1A). The top 15 up- and down-regulated DEGs were shown in the heatmap (Fig. 1B). After intersecting 200 hypoxia-related genes and 4171 DEGs, 45 DEHRGs were obtained for subsequently analysis (Fig. 1C).

### 3.2 Immune infiltrating cell analysis

**Fig. 2:**
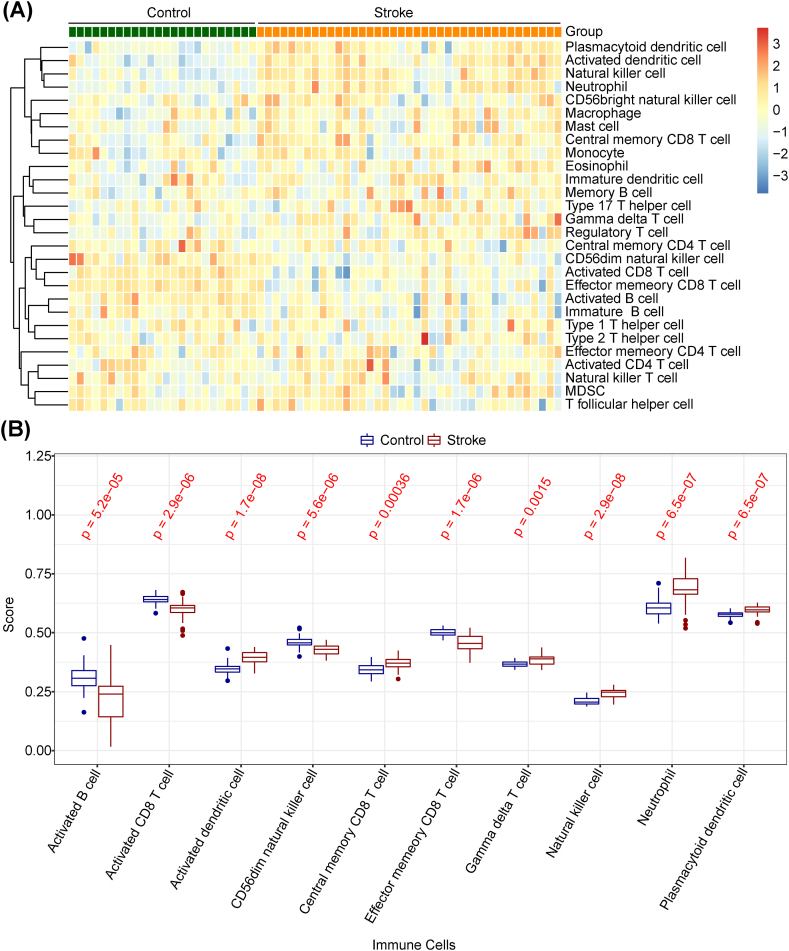
Identification of infiltrating immune cells in IS: (**a**) heatmap of 28 infiltrating immune cells in IS and normal sample, and (**b**) significantly different abundances of 10 infiltrating immune cells.

Since an existing study [10] focused on investigating the role of glial cell-mediated immune responses in the pathogenesis of IS, we initially explored the differences in immune cell infiltration between AD and normal samples by immune infiltration analysis. To acquire the differential immune cells between IS and control samples, the enrichment fraction of 28 immune infiltrating cells was depicted as a heatmap in Fig. 2A. The results in Fig. 2B showed that there were significant different abundances of activated CD8 T cells, activated B cells, CD56dim natural killer cells, activated dendritic cells, effector memory CD8 T cells, central memory CD8 T cells, natural killer cells, gamma delta T cells, neutrophils, and plasmacytoid dendritic cells between IS and control samples (adj.p < 0.05).

### 3.3 Construction of co-expression networks

**Fig. 3:**
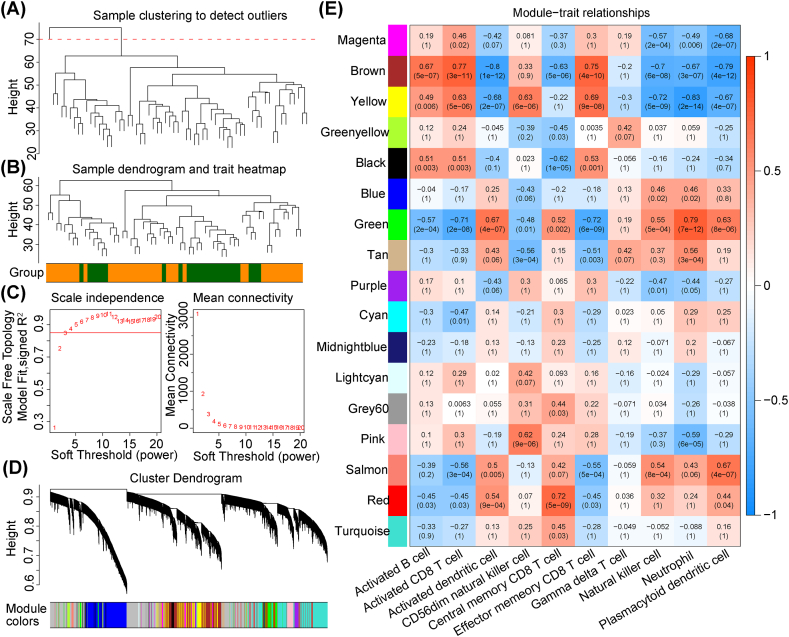
Development of co-expression networks: (**a,b**) detection of outlier sample detection (a) and clustering tree of samples (b); (**c**) calculation of the best soft-thresholding power; (**d**) idenrification of modules based on the co-expression network; and (**e**) relevance of modules and differential immune cells.

The above differential immune cells were used as clinical features to identify immune-related genes by WGCNA. Firstly, the samples in the GSE16561 dataset were clustered using the Euclidean distance of the expression to check for outliers. Fig. 3A indicated the presence of one abnormal samples. After removing the outliers, the remaining samples were clustered, in which green was the control sample and orange was IS sample. Next, when the soft threshold (power) was 4, R2 was 0.85, which meant connectivity tend to 0 (Fig. 3C). Afterthat, 17 modules were determined from the co-expression network by dynamic tree cutting algorithm (Fig. 3D). According to the module–trait relationships in Fig. 3E, we selected six modules (brown, yellow, green, pink, salmon, and red) with a correlation coefficient greater than 0.6 for further analysis. In total, 5426 immune-related genes in six modules were identified for subsequently analysis.

### 3.4 Identification of DEHRGs and functional enrichment analysis

**Fig. 4:**
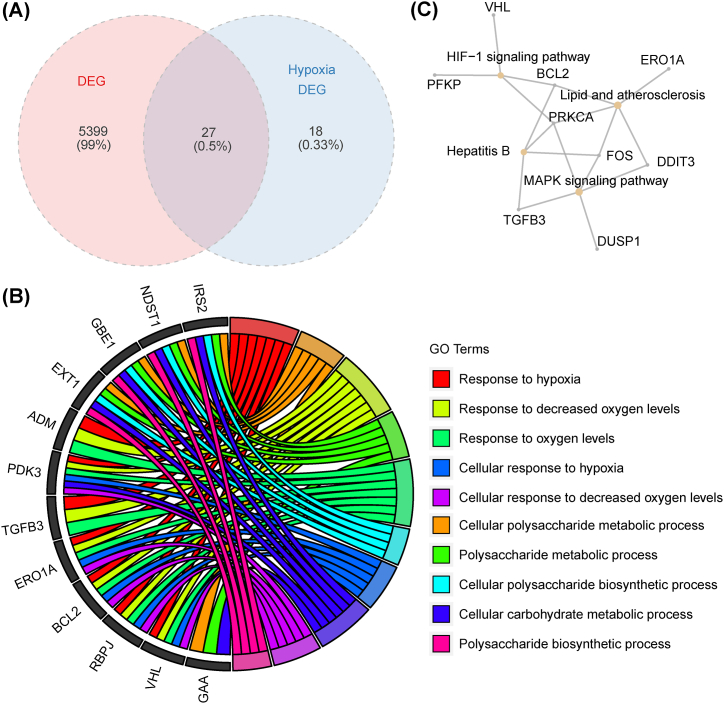
Identification of DEHRGs and functional enrichment analysis: (**a**) Wayne diagram of immune-related DEHRGs; (**b**) GO analysis of immune-related DEHRGs; and (**c**) KEGG analysis of immune-related DEHRGs.

We then took the intersection of the 45 DEHRGs and 5426 genes from the above modules, thus identifying 27 immune-related DEHRGs (Fig. 4A). GO and KEGG enrichment analyses were implemented to investigate the potential function of these immune-related DEHRGs. Fig. 4B illustrated that the GO analysis highlighted that immune-related DEHRGs were primarily enriched in response to hypoxia, cellular polysaccharide metabolic process, response to decreased oxygen levels, and polysaccharide metabolic process. As shown in Fig. 4C, the KEGG enrichment analysis pointed out that these DEHRGs were significantly included in lipid and atherosclerosis, as well as the HIF-1 signaling pathway. Therefore, we hypothesized that these 27 immune-related DEHRGs might be involved in the occurrence and development of IS through the above pathway.

### 3.5 PPI network creation and hub gene identification

**Fig. 5:**
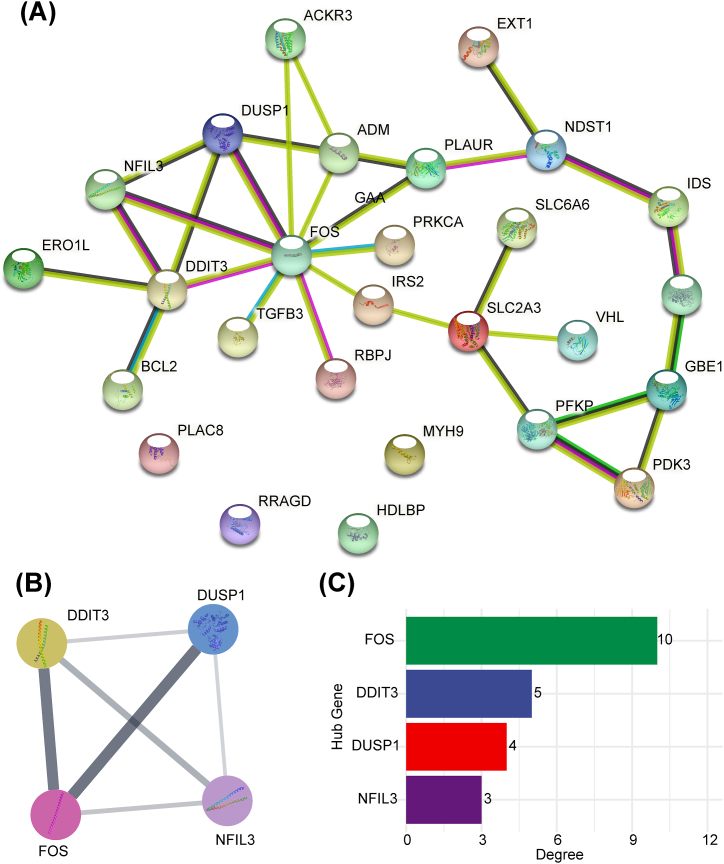
Identification of hub genes: (**a**) PPI network of immune-related DEHRGs; (**b**) MCODE plug-in identified hub genes; and (**c**) degree results for the four hub genes.

**Fig. 6:**
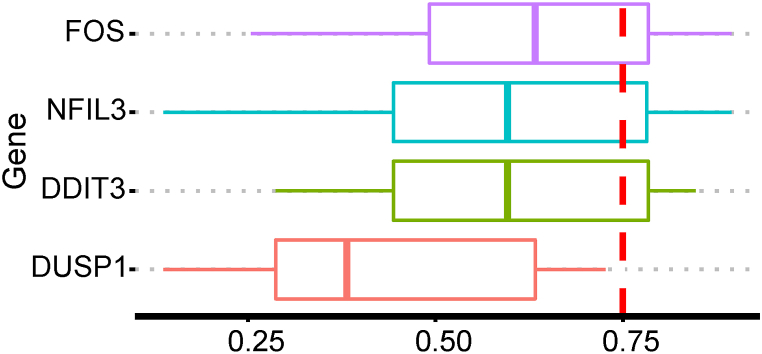
Functional similarity of the four hub genes.

**Fig. 7:**
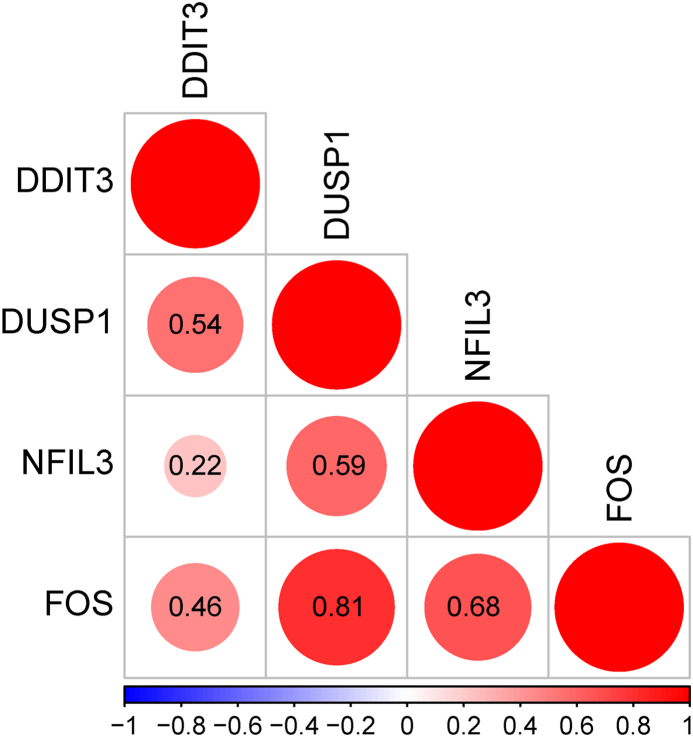
Correlation analysis of the four hub genes.

To explore the interaction among immune-related DEHRGs, the PPI network was established using the STRING database, in which there were complex interactions between genes other than PLAC8, RPAGD, HDLBP, and MYH9 (Fig. 5A). Subsequently, the above PPI network was imported into Cytoscape software and core module was selected using the MCODE plug-in to filter out hub genes. In total, four hub genes (FOS, DDIT3, DUSP1, and NFIL3) were determined for subsequently analysis (Fig. 5B). The degree of the hub genes was shown in Fig. 5C. The functional similarity findings revealed that FOS had the highest functional similarity score (Fig. 6). The correlation outcomes indicated that DUSP1 and FOS had the strongest positive correlation (r = 0.81; Fig. 7).

### 3.6 Correlation analysis among immune cells and hub genes

**Fig. 8:**
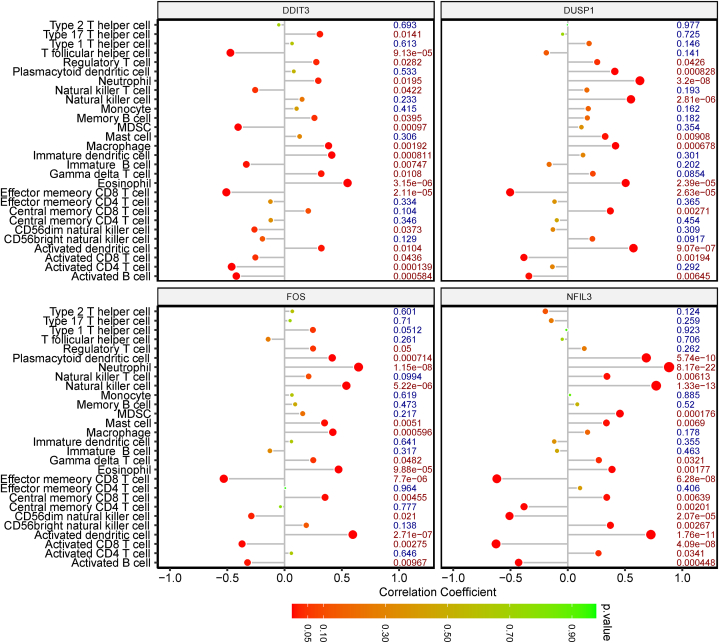
Correlation analysis between hub genes and immune infiltrating cells.

To determine the relationships between infiltrating immune cells and hub genes, we implemented correlation analysis. DDIT3 had the strongest positive link with eosinophils, as demonstrated by the findings; while DUSP1, FOS, and NFIL3 were most strongly positively associated with neutrophils. In addition, DDIT3, DUSP1, FOS, and NFIL3 were most strongly negatively associated with effector memory CD8 T cells (Fig. 8). Taken together with the results of the immune infiltration analysis above, we inferred that neutrophils and effector memory CD8 T cells played a role in the occurrence and development of IS, and that this might be related to the hub genes.

### 3.7 GSEA analysis

**Fig. 9:**
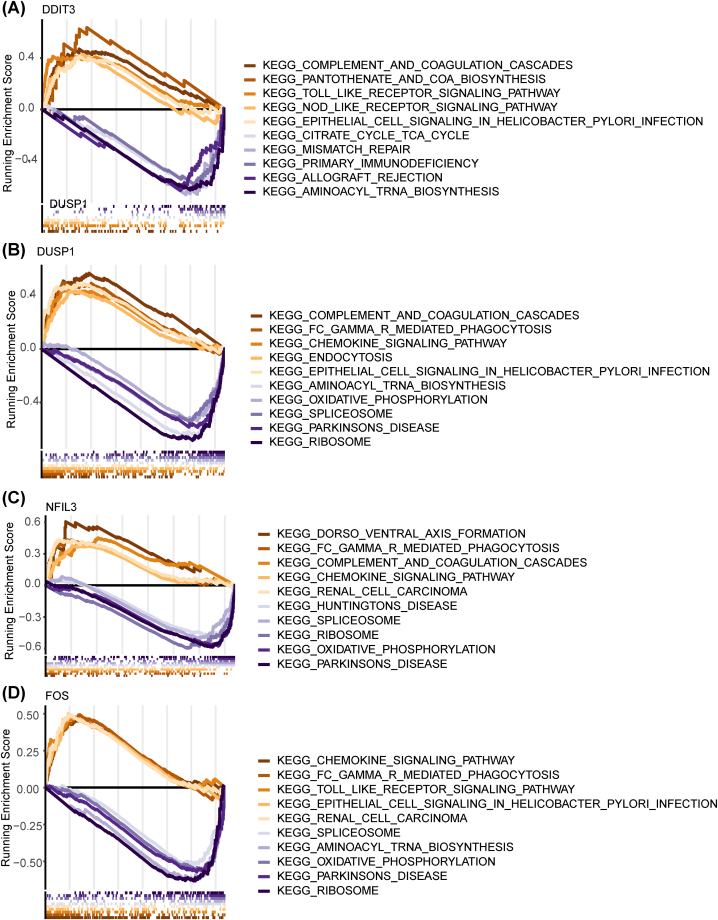
GSEA results related to the function of hub genes: (a) DDIT3; (b) DUSP1; (c) NFIL3; (d) FOS.

GSEA was employed to examine the roles of hub genes in IS (Fig. 9). Genes in the high-expression cohorts of DUSP1, FOS, and NFIL3 were all highly enriched in FC gamma R-mediated phagocytosis, and the chemokine signaling pathway. Genes in the high-expression cohorts of DDIT3 were greatly enriched in complement and coagulation cascades, pantothenate, and CoA biosynthesis. Genes in the low-expression cohorts of DUSP1, FOS, and NFIL3 were mainly enriched in oxidative phosphorylation, as well as Parkinson's disease. Genes in the low-expression cohorts of DDIT3 were highly enriched in allograft rejection and aminoacyl tRNA biosynthesis. Furthermore, there was a robust association between DUSP1 and the MAPK signaling pathway.

### 3.8 Screening of small molecule drugs

**Fig. 10:**
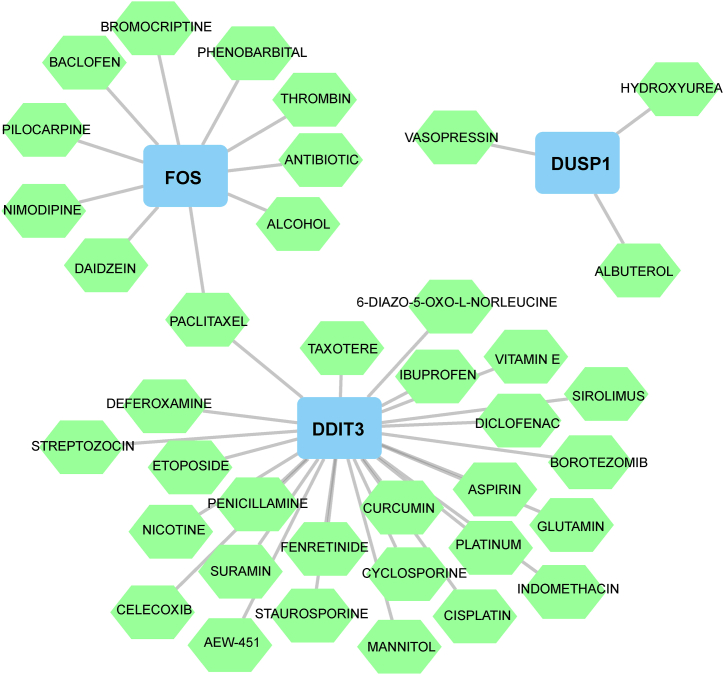
Drug–gene networks constructed by Cytoscape. Blue represents hub genes and green represents targeted drugs. (For interpretation of the references to colour in this figure legend, the reader is referred to the Web version of this article.)

For the treatment of IS patients, 39 potential drugs were determined using DGIdb (Supplementary Table 1),such as aspirin, suramin, Nimodipine, celecoxib, cyclosporine, vitamin E, deferoxamine, curcumin, and daidzein. In this study, 26, 10, and 3 drugs were found to interact with DDIT3, FOS, and DUSP1, respectively. Unfortunately, we were unable to locate any small-molecule drugs that might specifically target NFIL3 in this database. Furthermore, Cytoscape was used to create drug–gene networks, in which PACLITAXEL could act on FOS and DDIT3 (Fig. 10).

### 3.9 Prediction of potential miRNA–hub gene regulatory network

**Fig. 11:**
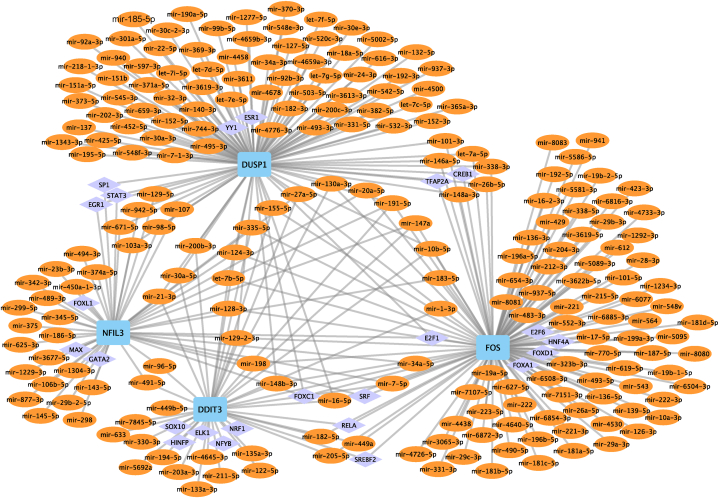
miRNA–hub gene–TF regulation network constructed using Cytoscape. The light blue rectangle is hub gene, orange oval is miRNA, and purple diamond is TF. (For interpretation of the references to colour in this figure legend, the reader is referred to the Web version of this article.)

To investigate the potential regulatory mechanisms of hub genes, we performed the prediction of TFs and miRNAs targeting the hub genes. Fig. 11 depicted the interaction network containing 36 TFs (such as, YY1, ESR1, ELK1, FOXC1, and SREBF2), 4 hub genes, and 278 miRNAs (e.g. miR-32-3p, miR-195-5p, miR-148b-3p, and miR-128-3p). Cytoscape was employed to construct a miRNA–hub gene–TF regulatory network for the purpose of comprehending the possible transcriptional regulation mechanism of hub genes (see Fig. 11). FOS was the hub gene that was controlled by the highest number of miRNAs (regulated by 105 miRNAs), as well as the highest number of TFs (regulated by 11 TFs).

### 3.10 Expression analysis and ROC curve analysis of hub genes

**Fig. 12:**
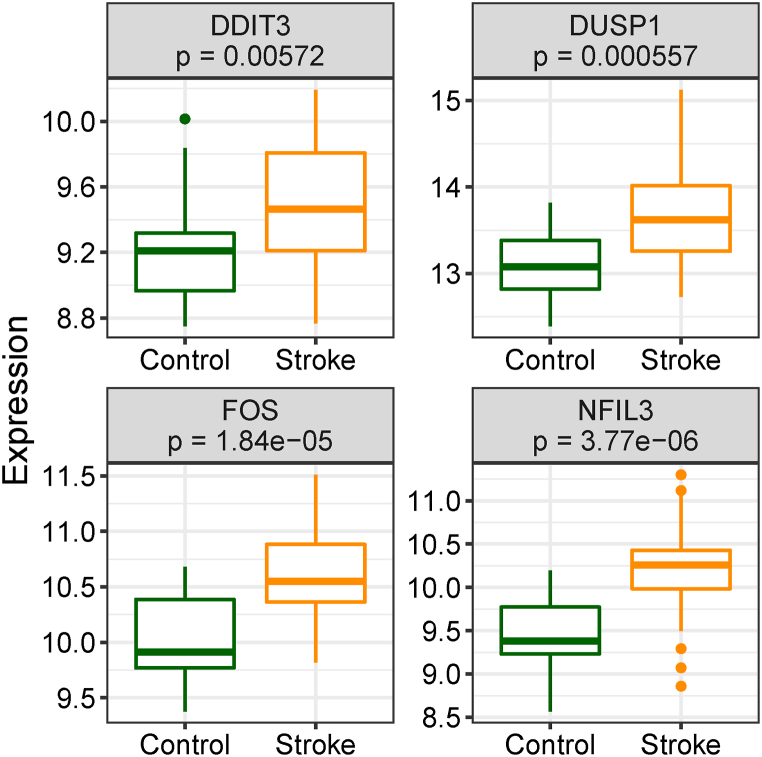
Expression analysis of the four hub genes.

**Fig. 13:**
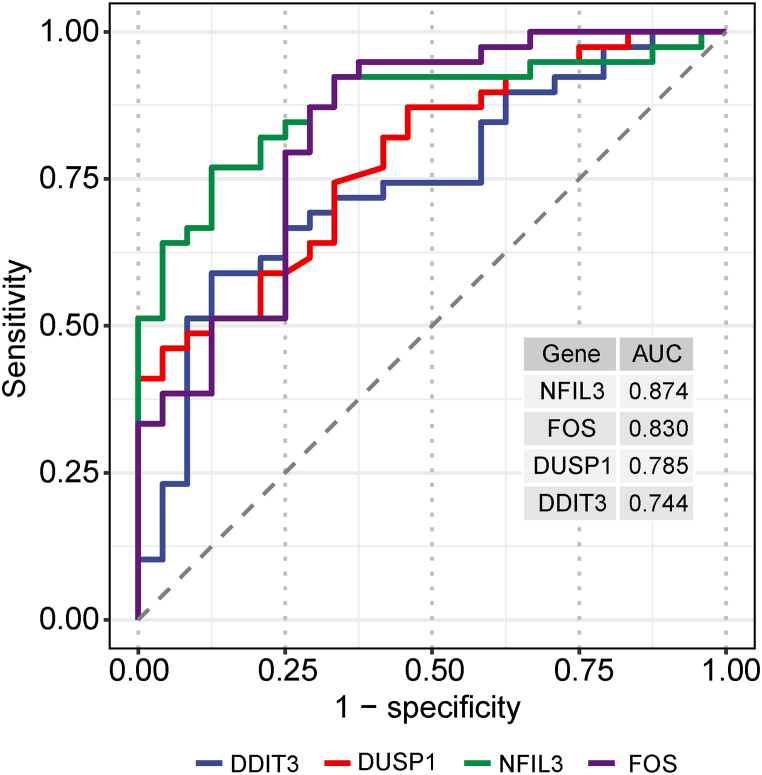
ROC curve analysis of the four hub genes. AUC, area under the curve.

We observed that the hub gene expression levels were considerably increased in IS samples, in contrast to those in the control group (Fig. 12). As shown in Fig. 13, the AUC values of FOS, DDIT3, DUSP1, and NFIL3 were 0.830, 0.744, 0.785, and 0.874, respectively, suggesting that these hub genes had good diagnostic values.

### 3.11 LASSO diagnostic model for IS established on the basis of the immune-related genes involved in IS

**Fig. 14:**
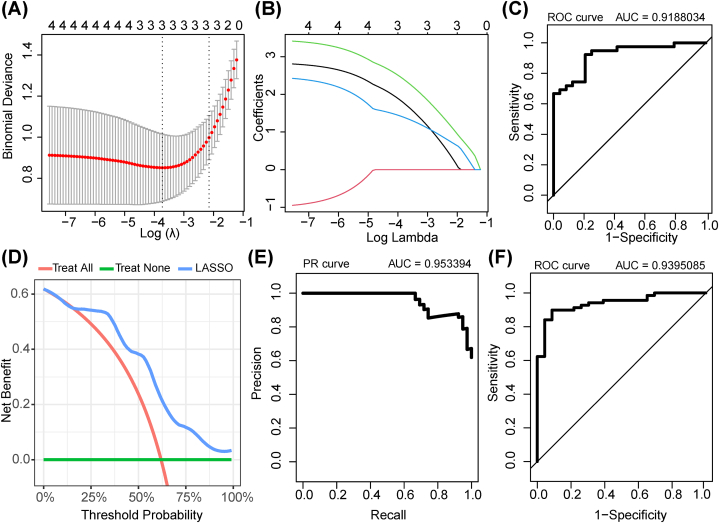
Construction and validation of the immune-related genes involved in IS diagnostic model: (**a,b**) The plot of error plots for 10-fold cross-validation (a) gene coefficients (b) and in LASSO analysis; (**c**) ROC curve of diagnostic model; (**d,e**) PR and DCA curves of diagnostic model; and (**f**) ROC curve of diagnostic model tested in GSE58294 dataset.

Thereafter, the LASSO algorithm was used to further screen the gene signature (DDIT3, NFIL3, and FOS) of IS from hub genes (Fig. 14A and B). Thus, we constructed a LASSO diagnostic model based on DDIT3, NFIL3, and FOS, and the ROC curve indicated that the model had high accuracy (AUC = 0.9188034; Fig. 14C). The PR and DCA curves further indicated that this model had good performance (Fig. 14D and E). Furthermore, we tested the LASSO diagnostic model on an external dataset (GSE58294). The ROC curve verified that it retained high diagnostic accuracy (AUC = 0.9395085; Fig. 14F). Compared with the other models (e.g., in an immune gene-related model), the AUC value of a previously described neural network diagnosis model was 0.909 in the training set, and 0.835 in the validation set [34]. Therefore, the immune-related DEHRG-related model constructed in this research presented better performance.

### 3.12 Expression verification of FOS, DDIT3, DUSP1, and NFIL3 in brain tissue after IS

**Fig. 15:**
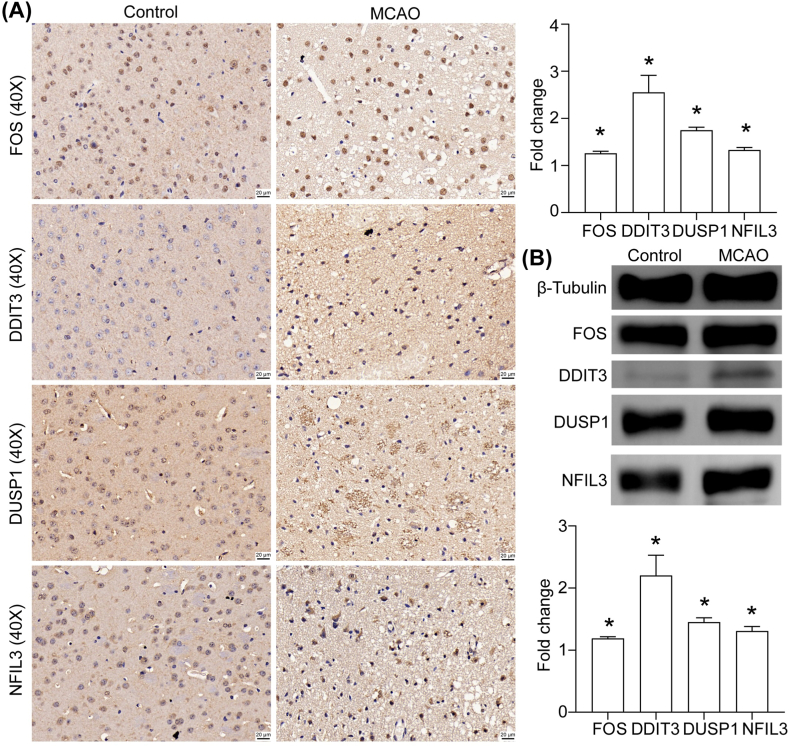
Expression verification of the four hub genes: (**a**) The expression of FOS, DDIT3, DUSP1, and NFIL3 after MCAO, observed by immunohistochemical staining; and (**b**) Expression of FOS, DDIT3, DUSP1, and NFIL3 after MCAO, examined by Western blotting; 3 mice/group, Scale bar = 20 μm. The entire data are expressed as means ± SDs. Fold change is the ratio of signal density between the control and MCAO samples. *P < 0.05 represents a statistically significant difference between the two groups. Each experiment was repeated three times.

An MCAO model in mice was used to construct the cerebral ischemic environment. The tissues of cerebral ischemic scars were taken for IHC staining and Western blotting, and the expressions of FOS, DDIT3, DUSP1 and NFIL3 after ischemia were detected. The findings revealed that the expression was increased, consistent with the results we obtained from the database analysis (Fig. 15A and B).

## 4 Discussion

IS can precipitate damage and dysfunction within the CNS, triggering the immune system into action. This rapid response contributes to neuroinflammation and secondary brain injury. The objective of this study is to investigate alterations in gene expression throughout the progression of IS, with the ultimate goal of identifying potential diagnostic biomarkers. We attempted to determine DEGs among normal and IS samples in this investigation. We first speculated that these potential DEGs might be a part of the disease progression of IS. Then, we constructed the PPI network and screened hypoxia- and immune-related hub genes as biomarkers. As a result, four key genes were observed and a diagnostic model that could accurately distinct IS was constructed. Finding biomarkers with good specificity and sensitivity is of great significance for patients with IS.

Using the ssGSEA algorithm, we determined that 10 kinds of immune infiltrating cells (activated CD8 T cells, activated B cells, CD56dim natural killer cells, activated dendritic cells, effector memory CD8 T cells, central memory CD8 T cells, natural killer cells, gamma delta T cells, neutrophils, and plasmacytoid dendritic cells) presented significant differences between IS and control samples, thus confirming that immune infiltrating cells play a crucial role in IS. Many studies have shown that activated B cells have a protective B-cell mechanism in the adaptive immune response of IS, thereby generating endogenous ischemic tolerance and, eventually, reducing injury from stroke and promoting repair [35]. There is evidence that T-lymphocytes also accumulate in the post-ischemic brain. Cytotoxic CD8^+^ T cells may induce brain injury through molecules released by their cytotoxic particles [36]. γδ T cells produce IL-17, which exacerbates ischemic damage [35]. It has been discovered that cells with dendritic cell (DC) attributes were present in the brains of the experimental animals after cerebral ischemia [36]. The most efficient antigen-presenting cells (APCs) are DCs, which are essential for both the adaptive and innate immunity [37]. NK cells have the ability to connect the nervous and immune systems in stroke patients. NK cells are closely related to post-stroke inflammation, immunosuppression, and infection. Excessive inflammatory reaction in the ischemic brain is one of the important causes for aggravating ischemic brain injury [37]. One of the first cells to respond to cerebral ischemia injury is the neutrophil, which produces inflammatory factors (e.g., ROS, MMP, and iNOS) [38]. Neutrophils are the most abundant type of white blood cells and play a critical role in the immune defense against infection. They are among the body's first line of defense and respond rapidly to infection or tissue damage. In the context of immune therapy, the role of neutrophils is complex as they contribute both to the resolution and progression of various diseases, including cancer, inflammatory conditions, and infections. The above results indicate that the immune cell infiltration results obtained in other IS studies were consistent with ours. In particular, DDIT3, DUSP1, FOS, and NFIL3 were significantly negatively correlated with effector memory CD8 T cells, suggesting that hub genes may affect the immune microenvironment of IS patients by regulating effector memory CD8 T cells.

After constructing the PPI network of hypoxia- and immune-related genes, four key hypoxia and immune genes (FOS, DDIT3, DUSP1, and NFIL3) were obtained. FOS proteins are implicated in the regulation of cellular processes such as proliferation, differentiation, and transformation. In certain instances, the expression of the FOS gene has been linked to apoptotic cell death. The FOS proto-oncogene can be induced in the brain as a result of cerebral ischemia [40]. Numerous investigations (both in vivo and in vitro) have shown that prolonged FOS induction precedes neuronal death following ischemia [41], and that FOS can facilitate the expression of pro-apoptotic genes resulting in cell death [42]. According to the most recent studies, the over-expressed AC079305/DUSP1 axis represents a stable and promising oxidative stress-related signature in IS [43]. The protein encoded by DUSP1 appears to play an important role in the human cellular response to environmental stress as well as in the negative regulation of cellular proliferation. FOS and DUSP1 have demonstrated high classification accuracy for strokes (AUC = 0.854) [39]. NFIL3 proteins are transcriptional regulator that binds as a homodimer to activating transcription factor sites in many cellular and viral promoters. Wang et al. have found that NFIL3 is a regulatory transcriptional factor that targets important genes involved in the cross-talk between immune suppression and stroke [40]. The DDIT3 proteins are implicated in adipogenesis and erythropoiesis, is activated by endoplasmic reticulum stress, and promotes apoptosis. There have been few reports on the relationship between DDIT3 and IS. These findings further demonstrated that up-regulation of FOS, DUSP1, and NFIL3 caused IS. We also found for the first time that overexpression of DDIT3 could increase the risk of IS, and we will continue to focus on this gene in the future.

GSEA was employed to analyze signal pathways linked to FOS, DDIT3, DUSP1, and NFIL3, and we found that they are related to inflammatory- and immune-related pathways. In various stroke models, STAT3 inhibition can lessen brain injury and enhance neurological effects [[41], [42], [43]]. Some studies have shown that activation of the STAT3 pathway after cerebral ischemia can change the microglial phenotype from pro-inflammatory to anti-inflammatory, partially improving brain injury [44,45]. Huang et al. have demonstrated that the FOS/IL-10/STAT3 pathway can play a neuroprotective role in cerebral ischemia [46]. Additionally, as previously reported, the activated STAT3/NFIL3 pathway and STAT3–NFkB/DDIT3/CEBPβ axis play regulatory roles in chemotherapy resistance [47,48], and lipopolysaccharide can induce the expression of DUSP1 and regulate macrophages through MAPK-STAT3 axis [49]. In our study, the KEGG enrichment analysis further highlighted a robust association between DUSP1 and the MAPK signaling pathway. Therefore, we speculate that FOS, DDIT3, DUSP1, and NFIL3 may regulate IS through the STAT3 pathway.

An miRNet method was used to construct and assess the target gene miRNA regulatory network and target gene-TF regulatory network of four hub genes. Many miRNAs related to the hub genes studied here were found to be closely related to IS. Long non-coding RNA intersectin 1-2 (lnc ITSN1-2) knockdown suppressed the inflammation and apoptosis in IS by regulating the miR-195-5p-mediated MAPK pathways [50]. By blocking the MAPK signaling pathway in IS, miR-137 prevents neuronal damage, inflammatory response, and oxidative stress [56]. Serum miR-32-3p, miR-148b-3p, miR-151b, let-7e-5p, miR-23b-3p, miR-29b-3p, miR-186-5p, miR-30a-5p, miR-124-3p, miR-128-3p, miR-192-5p, miR-222, miR-17-5p, and miR-221-3p may serve as potential diagnostic biomarkers for IS [[51], [52], [53], [54], [55], [56], [57], [58], [59], [60], [61], [62]]. Depleting sex-determining region Y-box 2 (SOX2) enhanced IS by the PVT1/miR-24-3p/STAT3 axis [63]. miR-335-5p, let-7b-5p, miR-34a-5p, and miR-3622b-5p have been reported as therapeutic targets or diagnostic biomarkers regarding IS treatment [[64], [65], [66], [67]]. 10.13039/100014337Furthermore, the TFs in our study have also been supported by the existing literature. Liu et al. have elucidate a neuroprotective role of inactivation of the YY1/DDIT4 axis in IS [68]. Fu et al. have suggested that a ESR1 polymorphism is significantly correlated with high IS risk [69]. Activation of NK cells in a SP1-dependent manner may provide a novel target for preventing post-stroke infection [70]. In addition, ELK1, FOXC1, and SREBF2 have been concluded to play a part in IS [[71], [72], [73]]. In this study, we also found that miR-32-3p and FOXC1 acted together on DUSP1 in the regulatory network to lead to IS by regulating the up-regulation of DUSP1 expression.Consequently, all of these TFs and miRNAs could serve as new biomarkers for IS.

In the event that you've experienced a stroke, your physician may prescribe medications, such as aspirin, as a preventative measure against a recurrence. Aspirin is a key component of a widely recognized treatment regimen for patients with a stroke history. Utilizing DGIdb, we pinpointed several potential pharmaceutical treatments for IS patients, including aspirin. Decades ago, studies suggested that suramin could diminish the infarction area post-stroke in animal trials [74]. Furthermore, a double-blind controlled study suggested that Nimodipine might offer therapeutic benefits in the management of acute stroke [75]. A population-based retrospective cohort study has shown that celecoxib reduce the risk of ischemic stroke in patients with rheumatoid arthritis in a dose- and time-dependent manner [76]. Relevant research also suggests that substances such as cyclosporine [77], vitamin E [78], deferoxamine [79], curcumin [80], and daidzein [81] may offer certain advantages in the prevention of ischemic stroke. The findings of our study demonstrated that Aspirin effectively targeted DDIT3, offering a novel insight into the specific mechanism underlying its therapeutic efficacy in treating IS.

In this study, we searched for different immune infiltrating cells and genes between IS and normal samples, investigated the diagnostic value of hypoxia- and immune-related genes, and speculated that these potentially differentially expressed genes may be the part of the disease progression of IS. The hub genes FOS, DDIT3, DUSP1, and NFIL3 were obtained as biomarkers with good specificity and sensitivity, which is of great significance for IS patients. A diagnostic model that can distinguish IS from normal samples with high accuracy was constructed, and the potential molecular mechanisms of key hypoxia- and immune-related genes regulating IS were discussed. Further exploration of these biomarkers might offer novel therapeutic and diagnostic targets for IS patients. However, our research is not without its limitations. Firstly, our findings are based on a limited sample analysis from the GEO database, making the expansion of the sample size a pressing matter. Moreover, we initially validated our results by constructing an MCAO mouse model. Despite the basic biological and genetic similarities shared between humans and mice, significant differences exist in their anatomical structures, metabolic pathways, and genomic compositions. These disparities may result in divergent responses to identical genes or biological processes between the two species. Secondly, due to the differing experimental conditions in mice and the physiological environment in humans, mouse models may not fully and accurately replicate human biological processes. Gathering a large number of clinical samples for experimental validation will effectively mitigate the impact of cross-species validation on research conclusions. Similarly, the efficacy and applicability of the targeted drugs for the hub gene we identified still require further validation in clinical practice. In conclusion, we will persist in exploring the value of these hub genes in IS and further investigate their mechanisms of action.

## 5 Conclusions

In conclusion, we discovered four distinct immune-related DEHRGs (FOS, DDIT3, DUSP1, and NFIL3) which, when combined with an interpretable machine learning method (i.e., the LASSO model), could successfully predict the development of IS. This LASSO model is expected to help aid clinicians in the diagnosis of IS. We offer novel diagnostic clues, which may facilitate personalized counseling for specific therapy.

## Ethics statement

The animal study was reviewed and approved by Fourth Military Medical University Committee on Animal Care (No. 20210419). All animal studies comply with the ARRIVE guidelines. No human studies are presented in this manuscript. No potentially identifiable human images or data are presented in this study.

## Funding

This work was supported by the Youth Nova Program of Shaanxi (no. 2021KJXX-19) and the 10.13039/501100001809National Natural Science Foundation of China (nos. 81871023, 82171458, 81771322, 82171363).

## Data availability statement

Publicly available data sets were examined in this study. This data can be found at the following addresses: https://www.ncbi.nlm.nih.gov/geo/query/acc.cgi?acc=GSE16561; https://www.ncbi.nlm.nih.gov/geo/query/acc.cgi?acc=GSE58294.

## CRediT authorship contribution statement

**Haofuzi Zhang:** Writing – review & editing, Writing – original draft, Visualization, Resources, Investigation, Formal analysis, Data curation, Conceptualization. **Jidong Sun:** Supervision, Data curation, Conceptualization. **Peng Zou:** Software, Investigation, Data curation. **Yutao Huang:** Writing – review & editing, Software, Methodology, Conceptualization. **Qiuzi Yang:** Software. **Zhuoyuan Zhang:** Conceptualization. **Peng Luo:** Writing – review & editing, Validation, Supervision, Project administration. **Xiaofan Jiang:** Writing – review & editing, Validation, Supervision, Funding acquisition.

## Declaration of competing interest

The authors declare that they have no known competing financial interests or personal relationships that could have appeared to influence the work reported in this paper.

## References

[1] Tadi P., Lui F. (2023). Acute stroke, in StatPearls. StatPearls Publishing Copyright © 2023, StatPearls Publishing LLC.: Treasure Island (FL) ineligible companies. Disclosure: Forshing Lui declares no relevant financial relationships with ineligible companies.

[2] Maida C.D. (2020). Neuroinflammatory mechanisms in ischemic stroke: focus on cardioembolic stroke, background, and therapeutic approaches. Int J Mol Sci.

[3] Collaborators G.B.D.L.R.o.S. (2018). Global, regional, and country-specific lifetime risks of stroke, 1990 and 2016. N Engl J Med.

[4] Katan M., Luft A. (2018). Global burden of stroke. Semin Neurol.

[5] Amarenco P. (2009). Classification of stroke subtypes. Cerebrovasc Dis.

[6] Donnan G.A. (2008). Stroke. Lancet.

[7] Donkor E.S. (2018).

[8] Sarfo F.S. (2018). Stroke among young west africans: evidence from the SIREN (stroke investigative research and educational network) large multisite case-control study. Stroke.

[9] Chamorro A. (2016). Neuroprotection in acute stroke: targeting excitotoxicity, oxidative and nitrosative stress, and inflammation. Lancet Neurol.

[10] Xu S. (2020). Glial cells: role of the immune response in ischemic stroke. Front Immunol.

[11] Pekny M. (2019). Astrocyte activation and reactive gliosis-A new target in stroke?. Neurosci Lett.

[12] Amantea D. (2015). Rational modulation of the innate immune system for neuroprotection in ischemic stroke. Front Neurosci.

[13] Azevedo P.N. (2020). Long-term changes in metabolic brain network drive memory impairments in rats following neonatal hypoxia-ischemia. Neurobiol Learn Mem.

[14] Kumari P. (2020). Fear memory is impaired in hypobaric hypoxia: role of synaptic plasticity and neuro-modulators in limbic region. Life Sci.

[15] George P.M., Steinberg G.K. (2015). Novel stroke therapeutics: unraveling stroke pathophysiology and its impact on clinical treatments. Neuron.

[16] Virani S.S. (2020). Heart disease and stroke statistics-2020 update: a report from the American heart association. Circulation.

[17] Wu Z., Zhang W., Kang Y.J. (2019). Copper affects the binding of HIF-1alpha to the critical motifs of its target genes. Metallomics.

[18] Barteczek P. (2017). Neuronal HIF-1alpha and HIF-2alpha deficiency improves neuronal survival and sensorimotor function in the early acute phase after ischemic stroke. J Cereb Blood Flow Metab.

[19] Dengler V.L., Galbraith M., Espinosa J.M. (2014). Transcriptional regulation by hypoxia inducible factors. Crit Rev Biochem Mol Biol.

[20] He Q. (2021). Biological functions and regulatory mechanisms of hypoxia-inducible factor-1alpha in ischemic stroke. Front Immunol.

[21] Xue L. (2017). Clinical significance of changes in serum neuroglobin and HIF-1alpha concentrations during the early-phase of acute ischemic stroke. J Neurol Sci.

[22] Engelhardt S. (2014). Hypoxia selectively disrupts brain microvascular endothelial tight junction complexes through a hypoxia-inducible factor-1 (HIF-1) dependent mechanism. J Cell Physiol.

[23] Wang X. (2017). Role of hypoxiainducible factor1alpha in autophagic cell death in microglial cells induced by hypoxia. Mol Med Rep.

[24] Ritchie M.E. (2015). Limma powers differential expression analyses for RNA-sequencing and microarray studies. Nucleic Acids Res.

[25] Chen H., Boutros P.C. (2011). VennDiagram: a package for the generation of highly-customizable Venn and Euler diagrams in R. BMC Bioinformatics.

[26] Hanzelmann S., Castelo R., Guinney J. (2013). GSVA: gene set variation analysis for microarray and RNA-seq data. BMC Bioinformatics.

[27] Langfelder P., Horvath S. (2008). WGCNA: an R package for weighted correlation network analysis. BMC Bioinformatics.

[28] Yu G. (2012). clusterProfiler: an R package for comparing biological themes among gene clusters. OMICS.

[29] Szklarczyk D. (2015). STRING v10: protein-protein interaction networks, integrated over the tree of life. Nucleic Acids Res.

[30] Yu G. (2010). GOSemSim: an R package for measuring semantic similarity among GO terms and gene products. Bioinformatics.

[31] Cotto K.C. (2018). DGIdb 3.0: a redesign and expansion of the drug-gene interaction database. Nucleic Acids Res.

[32] Fan Y., Xia J. (2018). miRNet-functional analysis and visual exploration of miRNA-target interactions in a network context. Methods Mol Biol.

[33] Robin X. (2011). pROC: an open-source package for R and S+ to analyze and compare ROC curves. BMC Bioinformatics.

[34] Liu W. (2022). Differential regulation of the immune system in peripheral blood following ischemic stroke. Biomed Res Int.

[35] Selvaraj U.M. (2016). Heterogeneity of B Cell functions in stroke-related risk, prevention, injury, and repair. Neurotherapeutics.

[36] Arumugam T.V., Granger D.N., Mattson M.P. (2005). Stroke and T-cells. Neuromolecular Med.

[37] Chen C. (2019). NK cells in cerebral ischemia. Biomed Pharmacother.

[38] Jian Z. (2019). The involvement and therapy target of immune cells after ischemic stroke. Front Immunol.

[39] Adamski M.G. (2014). Expression profile based gene clusters for ischemic stroke detection. Genomics.

[40] Wang X. (2022). Is immune suppression involved in the ischemic stroke? A study based on computational biology. Front Aging Neurosci.

[41] Rakers C. (2019). Stroke target identification guided by astrocyte transcriptome analysis. Glia.

[42] Chong H.C. (2014). Angiopoietin-like 4 stimulates STAT3-mediated iNOS expression and enhances angiogenesis to accelerate wound healing in diabetic mice. Mol Ther.

[43] Hristova M. (2016). Inhibition of Signal Transducer and Activator of Transcription 3 (STAT3) reduces neonatal hypoxic-ischaemic brain damage. J Neurochem.

[44] Chen S. (2017). Homocysteine exaggerates microglia activation and neuroinflammation through microglia localized STAT3 overactivation following ischemic stroke. J Neuroinflammation.

[45] Liu Z.J. (2019). Melatonin protects against ischemic stroke by modulating microglia/macrophage polarization toward anti-inflammatory phenotype through STAT3 pathway. CNS Neurosci Ther.

[46] Hua W. (2022). AKG attenuates cerebral ischemia-reperfusion injury through c-fos/IL-10/stat3 signaling pathway. Oxid Med Cell Longev.

[47] Peng Z. (2018). The STAT3/NFIL3 signaling axis-mediated chemotherapy resistance is reversed by Raddeanin A via inducing apoptosis in choriocarcinoma cells. J Cell Physiol.

[48] Canino C. (2015). A STAT3-NFkB/DDIT3/CEBPbeta axis modulates ALDH1A3 expression in chemoresistant cell subpopulations. Oncotarget.

[49] Bode J.G., Ehlting C., Haussinger D. (2012). The macrophage response towards LPS and its control through the p38(MAPK)-STAT3 axis. Cell Signal.

[50] Zhu F. (2021). LncRNA ITSN1-2 knockdown inhibits OGD/R-induced inflammation and apoptosis in mouse hippocampal neurons via sponging miR-195-5p. Neuroreport.

[51] Li P. (2015). Identification of circulating microRNAs as potential biomarkers for detecting acute ischemic stroke. Cell Mol Neurobiol.

[52] Cheng X. (2018). Exploring the potential value of miR-148b-3p, miR-151b and miR-27b-3p as biomarkers in acute ischemic stroke. Biosci Rep.

[53] Huang S. (2016). Identification of blood let-7e-5p as a biomarker for ischemic stroke. PLoS One.

[54] Wu J. (2017). Distinctive expression signatures of serum microRNAs in ischaemic stroke and transient ischaemic attack patients. Thromb Haemost.

[55] Wang R. (2018). miR-186-5p promotes apoptosis by targeting IGF-1 in SH-SY5Y OGD/R model. Int J Biol Sci.

[56] Wang W. (2018). Diagnosis of hyperacute and acute ischaemic stroke: the potential utility of exosomal MicroRNA-21-5p and MicroRNA-30a-5p. Cerebrovasc Dis.

[57] Qi Z. (2021). Serum extracellular vesicle-derived miR-124-3p as a diagnostic and predictive marker for early-stage acute ischemic stroke. Front Mol Biosci.

[58] Wang Q. (2021). Diagnostic and prognostic value of serum miR-9-5p and miR-128-3p levels in early-stage acute ischemic stroke. Clinics (Sao Paulo).

[59] He X.W. (2019). Increased plasma levels of miR-124-3p, miR-125b-5p and miR-192-5p are associated with outcomes in acute ischaemic stroke patients receiving thrombolysis. Atherosclerosis.

[60] Jin F., Xing J. (2017). Circulating pro-angiogenic and anti-angiogenic microRNA expressions in patients with acute ischemic stroke and their association with disease severity. Neurol Sci.

[61] Wu J., Du K., Lu X. (2015). Elevated expressions of serum miR-15a, miR-16, and miR-17-5p are associated with acute ischemic stroke. Int J Clin Exp Med.

[62] Cui Y. (2021). Diagnostic values of miR-221-3p in serum and cerebrospinal fluid for post-stroke depression and analysis of risk factors. Iran J Public Health.

[63] Chen Z. (2021). Depleting SOX2 improves ischemic stroke via lncRNA PVT1/microRNA-24-3p/STAT3 axis. Mol Med.

[64] Yang X. (2022). Study on potential differentially expressed genes in stroke by bioinformatics analysis. Neurol Sci.

[65] Chi N.F. (2020). Hyperglycemia-related FAS gene and hsa-let-7b-5p as markers of poor outcomes for ischaemic stroke. Eur J Neurol.

[66] Liang T.Y., Lou J.Y. (2016). Increased expression of mir-34a-5p and clinical association in acute ischemic stroke patients and in a rat model. Med Sci Monit.

[67] Zhu X. (2020). Uncovering the potential differentially expressed miRNAs and mRNAs in ischemic stroke based on integrated analysis in the gene expression Omnibus database. Eur Neurol.

[68] Liu H., Sun S., Liu B. (2021). Smurf2 exerts neuroprotective effects on cerebral ischemic injury. J Biol Chem.

[69] Fu R., Shen Y., Zheng J. (2019). Association between common genetic variants in ESR1 and stroke risk: a systematic review and meta-analysis. J Stroke Cerebrovasc Dis.

[70] Feng Y. (2021). miR-1224 contributes to ischemic stroke-mediated natural killer cell dysfunction by targeting Sp1 signaling. J Neuroinflammation.

[71] Zhang Q. (2019). Identification of key genes and upstream regulators in ischemic stroke. Brain Behav.

[72] He T. (2021). A novel SIRT6 activator ameliorates neuroinflammation and ischemic brain injury via EZH2/FOXC1 axis. Acta Pharm Sin B.

[73] Jin X. (2012). Association of sterol regulatory element-binding transcription factor gene polymorphisms with ischaemic stroke. J Int Med Res.

[74] Kharlamov A., Jones S.C., Kim D.K. (2002). Suramin reduces infarct volume in a model of focal brain ischemia in rats. Exp Brain Res.

[75] Paci A. (1989). Nimodipine in acute ischemic stroke: a double-blind controlled study. Acta Neurol Scand.

[76] Chen A.I. (2022). Celecoxib and Etoricoxib may reduce risk of ischemic stroke in patients with rheumatoid arthritis: a nationwide retrospective cohort study. Front Neurol.

[77] Nighoghossian N. (2015). Cyclosporine in acute ischemic stroke. Neurology.

[78] Loh H.C. (2021). Effects of vitamin E on stroke: a systematic review with meta-analysis and trial sequential analysis. Stroke Vasc Neurol.

[79] Selim M. (2009). Deferoxamine mesylate: a new hope for intracerebral hemorrhage: from bench to clinical trials. Stroke.

[80] Liu Z. (2017). Curcumin protects against ischemic stroke by titrating microglia/macrophage polarization. Front Aging Neurosci.

[81] Kim E. (2015). Daidzein augments cholesterol homeostasis via ApoE to promote functional recovery in chronic stroke. J Neurosci.

